# Appraising the Implementation of Complexity Approaches Within the Public Health Sector in Scotland. An Assessment Framework for Pre-Implementation Policy Evaluation

**DOI:** 10.3389/fpubh.2021.653588

**Published:** 2021-09-22

**Authors:** Claudia Zucca, Emily Long, Jeremy Hilton, Mark McCann

**Affiliations:** ^1^Jheronimus Academy of Data Science, Tilburg University, Tilburg, Netherlands; ^2^MRC/CSO Social and Public Health Sciences Unit (MRC), University of Glasgow, Glasgow, United Kingdom; ^3^School of Defence and Security, Cranfield University at the Defence Academy of the UK, Cranfield, United Kingdom

**Keywords:** health policy, population health management, complexity, systems theory, policy making, organizational behavior, normalization process theory, soft system methodology

## Abstract

Complexity approaches have gained international attention as potentially effective strategies to address population health challenges. In light of this, the Scottish government (Scot. Gov.) set the implementation of these approaches as the recommended practice for its public health sector organizations. This study evaluates the opportunity and feasibility of implementing complexity approaches in public health Scotland employees' everyday routine by employing a qualitative study that involves 20 stakeholders, representative of different organizations and roles. We made use of an assessment framework based on Soft Systems Methodology (SSm) and Normalization Process Theory (NPT) comprised of five phases: Phase One defines the boundaries, aims, and goals of the issue under study; Phase Two consists of data collection, drawing on the e-Health Implementation Toolkit (e-HIT); Phase Three involves short presentations and breakout group activities to provide information on the new policy; Phase Four employs system thinking tasks to structure debate and builds shared understanding among participants; Phase Five applies NPT to appraise the organizational position around complexity based on information from the preceding steps. We found two main obstacles to implementing complexity approaches: (1) a lack of a shared understanding of the key concepts in complexity and their practical implications; (2) stakeholders' fear of significant disruption to work routines and power relationships. We recommend addressing these issues with appropriate training and customization of goals and tools that may enable complexity approaches to succeed within the Scottish public health context. Our assessment framework allows the recognition of key mechanisms to support how Scotland's Public Health body can enhance the implementation of complexity approaches. The appraisal framework could be used to study early-stage policy implementation in other contexts.

## Engaging With the Complex Nature of Population Health

Most of the twentieth Century's population health challenges were addressed by analyzing the specific causes of health problems individually; an approach which can be deemed reductionist (1). After experiencing multiple failures in addressing social issues such as homelessness, substance use harms, or poverty, prominent scientists identified the need to frame problems as aspects of complex systems (2). In several countries around the globe, there are now health stakeholders such as practitioners, researchers, and governments advocating for a complexity approach to population health (2–5).

### The Scottish Context

The Scottish government (Scot. Gov.) outlined its intention to implement a “whole system approach” as part of its plan for public health reform (6–8). This study was initiated in the early stages of the reform program, before the organizations that now comprise Public Health Scotland merged into the new public health body. Under the pressure of a government-set priority, this study was designed to provide an appraisal of the feasibility and opportunity of implementing a complexity approach in the Scottish reform, aligned with the complexity approaches already utilized across the organizations. The current study is part of this synergic effort to improve the strategies and capabilities of the entire sector of public health.

### Complexity Approaches and Health Improvement

Due to its nature, the definition of complexity is not straightforward. We might call a system complicated when there are many elements and many relationships, but they can be unraveled and understood. However, we would consider a system as complex when the relationships between variables are so intertwined that they cannot be fully explained, leading to uncertainty between cause and effect (9, 10).

Complexity approaches are, at the same time, a way to frame reality, and a set of tools that allow us to understand and intervene in a system with inherent uncertainties. This composite nature of the concept is often expressed as a division between hard and soft approaches (11). While some authors focus on the advantages offered by computational and analytical complexity methods (5, 12–14), others point out that a complexity approach enables new organizational practices, which improve the working experience (15, 16). The interests of this second group of scholars widely overlap with Systems Theory (17). In this article, we consider complexity approaches as referring to both aspects.

Within population health, different applications of complexity have been applied in heterogeneous contexts. Complexity-related research in Canada and the UK has tended to focus on strategies to increase the use of complexity approaches (2, 18, 19), while in the US, Australia, and New Zealand, the focus has been on implementing complexity-based interventions (12, 20, 21).

Despite the differences in the conceptualization and application of complexity, there is a growing body of evidence around potential benefits (2, 20). However, to date, there is little information on the different forms of complexity that have been used and how successfully they have been implemented (22). Atkinson et al. (23) point out that complexity tools are already in use in many public health contexts; but we will never know how to enhance implementation until we research it.

Given the recent direction provided by the Scot. Gov. on best practices for public health sector, we developed an assessment framework for the appraisal of whether complexity approaches are feasible, opportune, and already employed in the public health community. Our research is the outcome of collaborative work that the research team and colleagues in Public Health organizations collectively developed.

## An Assessment Framework for the Appraisal of the Feasibility, Opportunity, and Extent of Normalization of a Potential Intervention

This section introduces our assessment framework for determining whether it is feasible and opportune to implement complexity methods among public health practitioners. The assessment framework integrates Normalization Process Theory (NPT), and Soft Systems Methodology (SSM). It also employs tasks that belong to the System Thinking Toolkit and draws upon a modified version of the e-Health Implementation Toolkit (e-HIT).

### Theoretical Framework

#### Normalization Process Theory

Normalization Process Theory is a sociological theory in the field of science and technology studies. It is a conceptual framework to evaluate how change is adopted, and the extent to which it becomes part of routine practice within a particular context. Normalization Process Theory can help us understand capacity and readiness for organizational learning. It is now widely used in health research to illustrate progress in the implementation of a new policy or technology (24–28). Normalization Process Theory is concerned with identifying and understanding four constructs: (1) how actors make sense of the work of implementing and integrating a complex intervention (i.e., Coherence); (2) how the actors engage with it (i.e., Cognitive Participation); (3) enact it (i.e., Collective Action); and (4) appraise its effects (i.e., Reflexive Monitoring) (29). The first two constructs relate to the phases preceding the adoption of an innovation. The latter two relate to the extent of engagement and involvement during implementation. Given that our study took place in the planning stage of complexity methods implementation, we considered only Coherence and Cognitive Participation, as we did not have information concerning the enactment or potential effects. More specifically, coherence describes the process of sense-making (29). Cognitive participation describes the process through which actors practically engage with a new policy (29).

#### Soft System Methodology

Soft System Methodology is an approach to organizational process modeling based on the soft system thinking theory (11). In order to make sense of a complex situation, it is established to frame it as a system of causal relationships among variables. Causality is often thought about in terms of the correlation between variables: e.g. “A” causes change in “B,” and is seen as a linear, directed process. However, when a system is complex, linear causal relationships are insufficient to explain the behavior that emerges. In those cases, approaches to understand complexity are more appropriate. The SSM approach is helpful when there are divergent views about the definition of a problematic situation. Due to the impossibility of providing a specific definition or boundary, we refer to a “soft problem” (11). SSM comprises four stages (30): (1) A problematical situation that needs to be addressed, (2) Purposeful fully flexible activities relevant to the situation need to be developed for each specific case, (3) A process of using the activities as devices to explore the situation and learn, (4) A structured debate about desirable and feasible change in the system under analysis. SSM provides tools for making sense of the specific issues, and fosters a process of information sharing and learning to agree on a specific definition of a problem. Assessing the feasibility and opportunity of implementing an intervention to establish complex approaches in the network of organizations providing health care services in Scotland is thus a soft problem.

#### e-Health Implementation Toolkit

e-Health Implementation Toolkit is a guide to help decide whether to embark on an implementation initiative (27). The toolkit consists of 21 statements that aid in evaluating the context of the intervention, its features, and the workforce response to the idea of the implementation. The evaluations are expressed on a scale from 0 to 10. Those scores provide a robust way to assess potential issues around how an innovation may be implemented into an organizational practice. The kit was originally applied in relation to e-Health interventions; we modified the phrasing of the 21 evaluation statements to refer to “complexity approaches” rather than e-Health.

#### System Thinking Tools

System thinking is an approach to complexity science that embraces a non-reductionist explanation of reality (31). System thinking promotes the use of several tools to disentangle, frame, and explain a problem as complex (11). Those tools are very different and tailored to the kind of problem addressed and the scope of the study. We employed a selection of these tools that foster a collective understanding of the employment of complexity methods among practitioners. The specific tools we used are explained in more detail in the assessment framework and results Section.

### Assessment Framework

The assessment framework introduced in this article comprises five phases. The first four phases correspond to the SSM four points introduced above, while the fifth consists of a final evaluation of the situation using the NPT construct as a benchmark.

Phase One specifies and defines the soft problem. It comprises a preliminary meeting with stakeholders to frame the problematic situation and set up the study, together with setting the focal issues and the aims.

Phase Two consists of the collection of preliminary data using the customized e-HIT toolkit. The collected information informs the design of two communal activities to make sense of the soft problem, and design tasks that foster a structured debate. Purposeful fully flexible tasks relevant to the soft problem need to be developed for each specific scenario during Phase Two.

In Phase Three, stakeholders participate in the two communal activities to make sense of the problem. The first activity involves the presentation of the first round of data collection, in order to be aware of differences and commonalities in their approach to the policy. The second activity provides training on the methods that the researchers setting up the study find crucial for making sense of the problematic situation and for understanding the policy domain. The choice of the methods needs to be compatible both with the goal of the project and with the resources available to the research team.

Phase Four employs an array of system thinking tasks to promote a structured debate on the extent to which the change that the policy would introduce is feasible and opportune.

Finally, during Phase Five, the collected information is assessed against the first two NPT constructs and their respective four components in order to evaluate the opportunity, feasibility, and state of usage of complexity approaches. The design is calibrated with the e-HIT toolkit design. A summary of the assessment framework is provided in Table 1.

**Table 1:** Outline of the underpinning theories, aims, and activities for the five phases assessment framework for the study.

**Theory behind each phase**	**Phases and aims**	**Tools in each phase**
SSM stage 1	**Phase One** Framing of the problematic situation together with stakeholders – Goals – Aims	Informal meetings
SSM stage 2	**Phase Two** Activity design informed by relevant data – First round of data collection – Activity design – Task design for Structured debate	Modified e-HIT toolkit System thinking theory
SSM stage 3	**Phase Three** Two activities to explore the situation and learn from each other – Presentation results modified e-HIT – Short seminars covering domain topics	Structured meetings
SSM stage 4	**Phase Four** Structured debate – System thinking tasks to foster a shared understanding	System thinking tasks during structured meetings
Normalization process theory	**Phase Five** Evaluation Appraisal of results against NPT Coherence and cognitive participation domains	Expert analysis

*SSM, soft system methodology*.

### Application of the Assessment Framework to our Study

#### Phase One

The first phase consisted of academics and stakeholders holding a series of consultations to assess the extent to which the Scot. Gov. directions concerning complexity approaches could be implemented into the organizational practice of the organizations designated to create Public Health Scotland. Together, they set the aims of the project and discussed the preliminary study set up.

#### Phase Two

The second phase involved the first round of data collection to inform the design of subsequent activities. We employed a version of the e-HIT modified with the phrasing appropriate for our study. The toolkit was complemented by three qualitative questions where respondents were asked to provide a definition of three concepts often discussed in relation to complexity and population health: “complexity,” “complex systems,” and “complex intervention.” We used individual in-person interviews to collect this information.

We then decided to have the two activities and the structured debate within the same setting, by organizing a workshop where stakeholders were asked to participate for a full day.

#### Phase Three

Phase Three involved two activities that we delivered in the morning of the workshop. We first presented the results of the interviews, promoting a concomitant discussion of the emerging themes. Afterward, we delivered three short seminars on domain topics. Namely, introductions to Systems mapping (i.e., qualitative/group model building elements), Social Network Analysis (SNA), and Agent Based Modeling (ABM).

The selection of these tools was made for two main reasons. First, this study aimed to enable practitioners to experience complexity tools that they would likely employ as part of the Scot. Gov. reform. The type of activities practitioners carry out in their organizations could be facilitated by employing the methods that we selected. System mapping offers qualitative tools to ensure that each stakeholder is able to contribute equally. Network analysis provides a set of qualitative and quantitative tools that assist practitioners to make sense of group behavior (e.g., observing how people respond to an intervention). Agent-based modeling advances the two previous approaches by enabling practitioners to predict the possible outcome of health interventions. Second, since methods relevant to complexity require high-level expertise, we relied on the knowledge within the collaborating institutions to support their implementation. Researchers in our group are experts in the domains of system mapping, network analysis, and agent-based modeling.

Three academics from the University of Glasgow (UoG) (including two co-authors) delivered the three seminars. During the presentations, the workshop attendees completed SWOT charts (32) to collect their thoughts on the methods presentations.

#### Phase Four

Phase Four involved structured activities to facilitate a shared understanding of the desirable and feasible changes related to the use of complexity methods. We employed five system thinking tasks: (1) Post-it sorting (33), (2) Pig in the Middle diagrams (33), (3) Context Diagrams (34), (4) Laddering (34), and (5) Six Cohering Questions (derived from CATWOE [(35), p.23–4].

These tasks were performed within the context of the world café method (36), which mingles the groups of participants to foster more information-sharing and discussion.

#### Phase Five

Information collected through Phases Two, Three, and Four was analyzed thematically (37), and assessed against the two NPT constructs introduced above. The research team gave an appraisal of each component having reached a low, moderate, or high level. This evaluation provided a descriptive summary of the factors which related to the ease of adoption of complexity methods.

## Results

Twenty key stakeholders, five academics, and 15 employees from public health organizations took part in this study^1^. They represented five different organizations, with several of them covering managerial roles. The largest group worked for National Health Service Health Scotland (NHS HS), the organization with a role around health improvement. Two stakeholders were from the Information Service Division (ISD, data management, analytics, and intelligence), one of whom also worked with NHS HS. The five academics worked for the UoG and collaborated together. Two respondents worked for Health Protection Scotland (HPS, risk, incident, and disease outbreak management) and one for the Scot. Gov.

**Figure 1:**
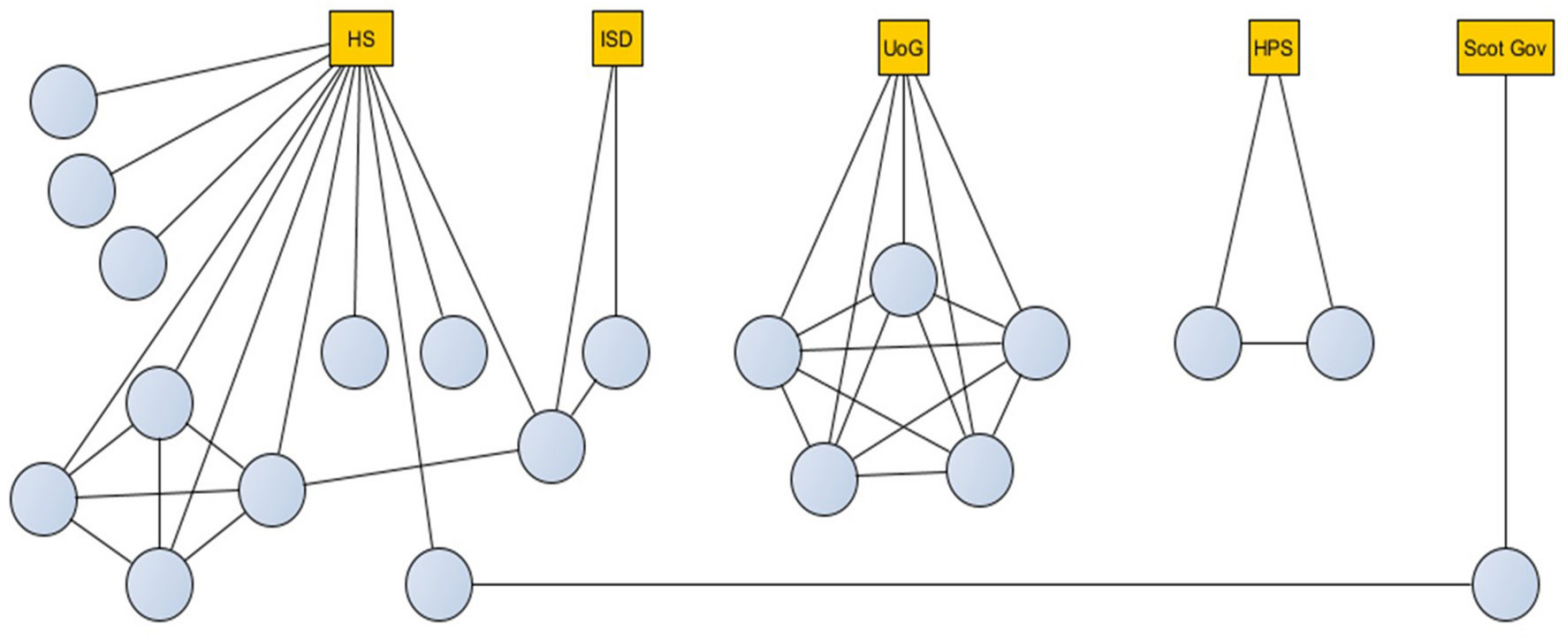
Collaboration and organizational affiliation network for 20 interview and workshop participants. HS, Health Scotland; ISD, Information Service Division; UoG, University of Glasgow; HPS, Health Protection Scotland; Scot Gov, Scottish Government.

Figure 1 illustrates the network of collaborations and organizational affiliations. Blue dots represent stakeholders, while yellow squares represent the organization for which they work. Links represent co-working. Gender was evenly represented in the sample (8 women, 12 men).

The interviews we carried out within Phase Two, that took place between March and May 2019. Participants convened together for a workshop on the 4th of June for 6 h of scheduled activities (Phase Three in the morning, Phase Four after Lunch).

### Phase Two Findings

#### Qualitative Questions Within the Individual Interviews

Participants were asked to provide a definition of three domain topics: “complexity,” “complex systems,” and “complex intervention.” Two categories of responses emerged from the questions around understanding of complexity, reflecting the division in the literature around the two main areas of complexity approaches, which we termed “organizational” and “methodological” approaches. Eight stakeholders provided explanations focused on the organizational advancements offered by complexity theories, while seven stakeholders suggested definitions in line with complexity methods. The five academics belonged to the second group.

Organizational approaches are in line with soft-systems theories, and eight stakeholders focused on its social and collaborative side. They wanted to promote the use of techniques such as system thinking and system mapping in the everyday work of their respective organizations. They viewed complexity approaches as a way to improve organizational efficiency and foster a more pleasant and inclusive working environment.

On the other side, the 12 respondents (stakeholders and academics) that defined complexity approaches as methods, were interested in the potential of complexity science to understand social reality through data collection and artificial reality simulation. They wanted to integrate analytical tools from complexity science into the theoretical and analytical work.

The definition of complex system reflected the same division. The group conceiving complexity as tools to improve working life understood complex systems as systems of social relationships. The respondents conceiving complexity as a set of methods, defined complex systems as non-linear interaction between agents. There was an agreement between the two groups in the definition of a complex intervention, which is a term commonly used in health intervention research, but often without reference to a broader definition of complexity.

Generally speaking, respondents found it hard to talk about complexity. We had a few “*I don't know”* answers, and one person said, “*this is difficult, I should have prepared.”* Two respondents stated that the methodology they already employ with their teams is not any different from complexity.

#### Modified e-Health Implementation Toolkit

**Figure 2:**
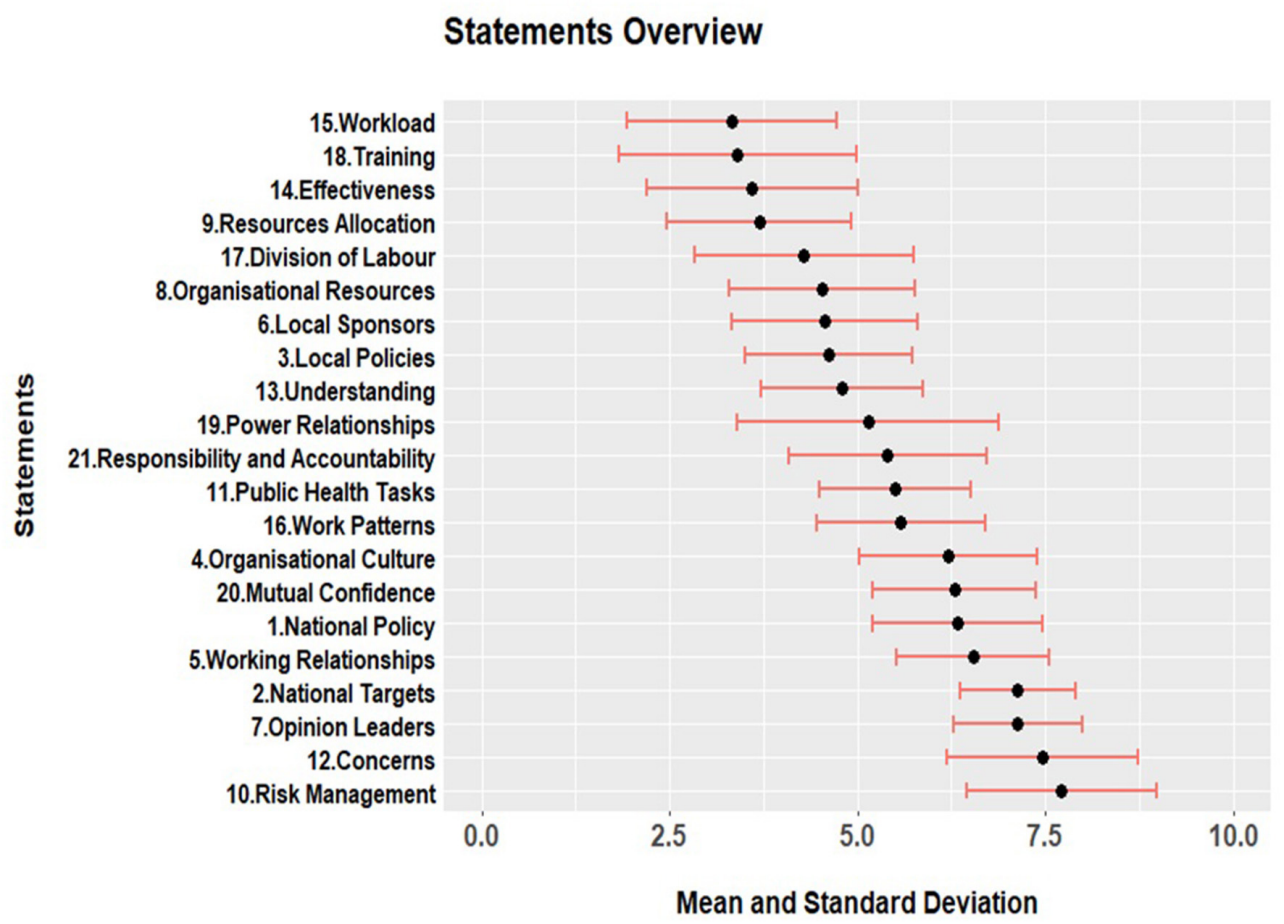
Mean and standard deviation for positive or negative appraisal of complexity approaches in relation to 21 topics. Higher numbers represent positive appraisal.

Given the small sample size, and the fact that e-HIT ratings do not benefit from a validated scale, we interpreted these results by plotting the mean and standard deviation of the ratings and qualitatively appraising the figure. Figure 2 shows the mean and the standard deviation for each statement employed in the modified e-HIT. Keywords on the left-hand side provide a reference to the statements (See the Supplementary Material for the full statement list). Higher values represent a more positive evaluation.

A visual examination of figure two shows that most scores are in the middle range values with a significant standard deviation. Overall, the respondents showed moderately positive responses, but there was high variation (i.e., respondents were rarely in strong agreement on any of the answers).

However, we can better understand the results if we examine the five clusters of statements that naturally form. On average, the areas of most significant concern (i.e., that reported the lowest average scores—between 2.5 and 3.7) refer to the training required for the implementation (s18), the increase in workload that this would entail (s15), the effectiveness of complexity methods for achieving goals (s14), and the changes in resource allocation (s9).

The second group includes statements with an average score between 3.9 and 5. The themes in this group concerned changes to the current division of labor (s17), concerns around under-resourcing (s8), the appropriateness of complexity methods to address local policies (s3), and uncertainty in the understanding of how to use complexity in practice (s13).

The third group includes more positive statements that score on average between 5 and 5.5 concerning compatibility with existing power relationships (s19), the alignment between responsibility and accountability (s21), the suitability of complexity for public health tasks (s11), and making work more efficient (s16).

Group number four had more positive ratings with an average score between 6.7 and 7. It includes whether respondents perceive complexity methods as aligning with national policy (s1), receptiveness of the organizational culture to change (s4), pre-existing co-operative relationships (s5), and the potential to increase confidence between groups in each other's work (s20).

Finally, the fifth group of statements scored between 7.3 and 7.6. These are the most positively evaluated areas suggesting that respondents do not think there are particular concerns for the adoption of complexity methods (s12) or risks (s10) in using them, or that opinion leaders would oppose their implementation (s7), or that they would interfere with the achievement of national targets (s2).

The overall evaluation suggested that participants are favorable toward employing complexity methods, but at the same time, skeptical. They liked the idea, but they did not fully understand it, and they have trouble envisioning a possible new scenario and how it would fit into their work without additional resources and support.

### Phase Three Findings

The SWOT chart task is aimed at helping participants understand strengths, weaknesses, opportunities, and threats posed by each method presented during the short seminars during Phase Three. To clarify, if we think of a method such as participatory observation, one strength could be that is very well-established, but a weakness could be that it requires particular abilities from the researcher that make the outcome vary consistently. At the same time, the possibility of collecting very detailed information on participants could be an opportunity, while the risk of having research that is very specific and not any more comparable is a possible threat posed by the method. This structured way of observing pros and cons of an innovation provide us with an overview of the methods that are considered domain topics in this new policy. Overall, SNA was the method more positively evaluated by respondents since it reported the highest ratio between positive and negative comments (See Supplementary Material for details).

The thematic analysis of the SWOT charts found that the participants viewed a range of positive aspects that varied according to the approach. The overall appraisal of SNA and ABM is very much comparable since both are perceived as hard methods that can improve the understanding of causality in systems and potentially enrich statistical toolkits. Their negative sides reside on the difficulties using them, and the risk of misuse due to the high level of abstraction they might reach, assumptions made in the models, and the required expertise to apply them. The appraisal of system thinking shows that it is perceived as a method that can improve organizational practice facilitating collective work. The negative points concern its feasibility for actual research tasks. There was a higher level of commonalities around the negative themes, the potential for inappropriate use, and difficulties in implementing the methods and their usability.

### Phase Four Findings

Phase Four engaged participants with system thinking tasks designed to structure debate by performing collaborative activities. They were assigned to one of four thematic tables according to expertise, and worked on the first three system thinking tasks (i.e., post-it sorting, pig in the middle diagram, context diagram). Table seating was then shuffled according to the world café method. During the second table configuration, the new groups reviewed and extended the previous table's work, and completed new tasks (i.e., Laddering, and Six Cohering Questions). One table was assigned to the discussion of the introduction of complexity approaches in the new public health body, the other three tables were assigned each of the methods presented: SNA, ABM, and Systems mapping.

#### Tasks in the First Table Configuration

There was a considerable level of engagement with the post-it sort, evidenced by the large number of post-its produced. The post-its provided unstructured comments, criticisms, and suggestions and were placed on a board so that participants could read each other's impressions during coffee breaks.

To complete the Pig in the Middle Diagram, participants were asked to place a sheet of easel paper on their table with the topic area of their group written in the middle, and then connect all the interested stakeholders, alongside the reason for their interest, to the written topic label with an arrow. The context diagram (i.e., the third system thinking task), built on the pig diagram by asking participants to focus on one stakeholder from the previous task, and classify the potential actions of this stakeholder under four categories: (1) factors under the stakeholder control, (2) factors the stakeholder can influence, but are not under direct control, (3) immediate environment that constrains stakeholder's behaviors and choices, and (4) the wider context that influences what does or does not occur.

Thematic analysis of the Pig in the Middle and the Context Diagrams (reported in the Supplementary Material) shows consistency across tables: the same seven key topics were raised by participants independently from their assigned table topic. The relevant stakeholders that are directly involved in the usage of complexity approaches are: (1) private actors, (2) government and policymakers, (3) other agencies and networks of practitioners, (4) public health actors, (5) the Scottish population, (6) experts, and (7) stakeholders implementing interventions. The reasons and motivations around why these actors may engage with complexity approaches differed across each group theme (see the Supplementary Material), according to the stakeholder chosen for the context diagram and the role played by the focal stakeholder.

#### Tasks in the Second Table Configuration

In the laddering task, participants were asked to choose a particular empirical perspective on the topic of their table. By asking why and how they would focus on specific tasks rather than others, the activity helped them to move from abstract ideas to developing strategies for the implementation. The SNA group decided to focus on the use of complexity methods to reduce inequalities in Scotland. The ABM group focused on the role played by local authorities to develop open space strategies. The system mapping group focused on strategies to reduce obesity in Glasgow. The HS Scotland group chose the analysis of the role of Research Development Groups to improve system practice in Scotland.

The comparison between the output of ladders across groups shows the different conceptualization of these novel methods each group has. Those differences mirror different backgrounds and interest of participants, translating to different expectations from the new proposed policy. A cross-cutting theme was enhancing collaboration among practitioners and institutions, while groups varied in reflecting on the methodological implications and moral justifications of adopting new practices.

We employed the six cohering questions task to promote the emergence around consensus of purpose for implementing a complexity approach, and a set of actions that the group found useful next steps to achieve that purpose. It also promoted clarity over who is involved in delivery, oversight, and the broader constraining factors that must be taken into consideration.

There was considerable variability between how the groups performed this task. The SNA group focused on how SNA could be applied to understanding inter-organizational collaborations. They produced a detailed set of answers around how to implement a future SNA research study.

The ABM and System Mapping tables also developed a set of actions to encourage the use of systems methods in PHS, but some gaps remained. One table was uncertain about who would be involved in implementing the changes, while the other did not outline the wider beneficiaries, sponsors, and constraints.

The table looking at how systems practice could be more broadly implemented in Public Health Scotland, did not complete the stages of the task. Instead, this group talked in more general terms about the challenges faced, but failing to reach final agreement on goals.

## Discussion

Within the assessment framework we employed, Phase Five assessed the collected data against the first two constructs of NPT (Coherence, and Cognitive Participation) to evaluate the feasibility and opportunity of complexity approaches in the practice of the public health sector in Scotland. Coherence analyses the creation of shared meaning that it is necessary to introduce change for a group of people that need to understand each other and work together. It is not feasible to introduce innovation that the involved parties cannot understand (29). Cognitive Participation assesses the extent to which stakeholders want to engage with the innovation. If they show a high level of engagement, this may reflect the opportunities for the use of a novel approach that could be easily accepted within the organization (29).

The NPT constructs are subdivided into four components each. We appraised the fulfillment of these subdomains as high, moderate or low level. We presented the results below complemented with a summary in Table 2.

**Table 2:** Appraisal of the level of each NPT constructs based on a summative evaluation of the data from the project (Phase Five summary).

**Coherence (Sense making)**	**Cognitive participation (Engagement)**
**Differentiation**	**Initiation**
High	Low
**Communal specification**	**Enrollment**
Moderate	Low
**Individual specification**	**Legitimation**
Moderate	Moderate
**Internalization**	**Activation**
Moderate	Low

### Coherence (Sense Making)

#### Differentiation

We evaluated the extent to which actors understood how the innovation and its constituent parts were different from each other (29). The data collected during Phase Two, three, and four suggests that participants can make a clear differentiation between complexity approaches and current organizational practice in the majority of the situations. Our summary is that complexity is highly differentiated from existing practice (Table 2).

#### Communal Specification

We evaluated the extent to which actors would work together to build a shared understanding of the aims, objectives, and expected benefits of the innovation (29). During Phase Four, stakeholders showed interest in working together and compared different approaches to complexity. However, they were not entirely open to shift to a common ground since they were still very attached to their viewpoints based on their previous experience in the field. More work needs to be carried out to acquire a full communal specification, which at this stage we deemed as moderate.

#### Individual Specification

We evaluated the extent to which actors understand their specific tasks and responsibilities around the innovation (29). During Phase Four, participants had the opportunity to approach their current work using complexity approaches (Laddering and Six Questions soft systems tasks). However, this experience is limited, and it engaged certain people with higher natural propensity more than others. We appraised this component as a moderate level.

#### Internalization

We evaluated the extent to which actors understand the value, benefits, and importance of the innovation (29). This component is assessed with the SWOT chart in Phase Three and the tasks in Phase Four. In general, participants understood the benefits and importance of the innovation, but there were many concerns and negative views of the methods, which must be addressed in order for the methods to be used successfully. For these reasons, internalization achieved a moderate level.

### Cognitive Participation

#### Initiation

We evaluated the extent to which actors would work to drive the innovation forward (29).

Key concerns identified with the modified e-HIT include: need for training, increase in workload, effectiveness of the methods to meet public health goals, and changes in resource allocation.

Even if we observed a general interest in the complexity approaches, there is resistance and uncertainty around how their use will affect the organizational practice. The participants perceive complexity as something that would introduce a significant change in their working life. Our sample largely consisted of senior employees in their respective organizations, and their position might imply a higher level of resistance to change since they are used to managing their staff in a way that might be modified by the introduction of complexity approaches. Their centrality in the organization itself might be compromised, too, since complexity suggests less hierarchical working practices.

The group that discussed implementing complexity approaches in Public Health Scotland experienced the most difficulty performing the Six Cohering Questions task. This suggests that workshop delegates were not positive toward the adoption of complexity methods at the time, or at least that they did not agree among themselves on how to proceed with implementation. For these reasons, the initiation component was evaluated as low.

#### Enrollment

We evaluated the extent to which actors would organize or reorganize themselves and others to collectively contribute to the work involved in the innovation (29). The modified e-HIT uncovered further concerns around: division of labor, allocation of organizational resources, the appropriateness of the new approach to address local policies as an organization, and uncertainty in the application of complexity in their everyday work. While Initiation was more concerned with the individual level, enrolment focused more on the organizational side. Again, even if actors were, to a certain extent, positive toward the idea, they were afraid of facing significant changes in their routines. These concerns would prevent them from a collective contribution to the success of the innovation. For this reason, this component was rated at a low level. The implementation should provide incentives for stakeholders to collaborate with the new rules and start a new organizational practice.

#### Legitimation

We evaluated the extent to which actors would work to ensure that other participants believe it is right for them to be involved and that they can make a valid contribution (29). During the workshop, participants worked together to achieve the common goal of rethinking their usual practices with new points of view involving new practices. If the intervention aimed at introducing Complexity methods would provide an incentive to support Initiation and Enrollment so that actors do not feel threatened by the innovation, evidence from the workshop shows that they would work to ensure that other participants are fully involved and able to contribute. Even if the engagement with the activities shows a positive attitude within the domain of this construct, it remains a margin of uncertainty that is reflected by this domain being rated as moderate.

#### Activation

We evaluated the extent to which actors collectively defined the actions and procedures needed to sustain a practice and to stay involved (29). During the workshop, participants outlined practical steps and actors involved in using complexity approaches for public health tasks. With the Pig diagram and the Context diagram tasks, participants engaged with identifying the boundaries of their and others' involvement with their respective domain. The fact that the results of these tasks overlapped across stakeholder tables shows a strong foundation for defining the domain of required action. However, the lack of a shared definition of complexity approaches and the general uncertainty on the magnitude of the changes the organization would face in case of adoption, prevented participants from a full engagement with the definition of actions and procedures to sustain the innovation. It's difficult to fully assess this construct, as the participants were speculating rather than carrying out sustained action. It's still possible that any new approach within Public Health may not be sustained. As such, the component was appraised as low.

### Phase Five Overall Evaluation

Coherence's components are evaluated with a majority of moderate and one high, denoting that the people involved in the study have a moderately clear picture of what introducing complexity methods into the practice of Public Health Scotland would entail. However, Cognitive participation's components are evaluated with a majority of low and one moderate. Results demonstrate that participants are not convinced that the new policy would work in their environment, and are not supportive of the adoption in the current conditions.

The participants did not arrive at a shared understanding of what complexity approaches are, and what using them entails. Even if this outcome would have been desirable from a policy-making perspective, it does not affect this research, which is simply aimed at appraising the level of understanding and informing policy makers accordingly. Given that the participants were more senior and well-engaged with the policy direction, it is very likely that the wider organization will have similarly diverse understanding or low understanding. This can be addressed with capacity-building work, training, and development, such as focus groups that work together for the intervention design and involve their respective colleagues in the group where they belong.

Overall, the moderate levels of sense-making of complexity approaches suggests that the wider organization could achieve higher coherence with an appropriate program of training activities. For example, an introduction to the concepts and training in applied methods, accompanied by plans to identify how to match appropriate methods to the respective functions and teams within Public Health Scotland.

The results showed concerns around disruption to working routines in relation to active engagement, and the opportunities to implement complexity approaches. Implementation plans can address this problem by ensuring that a suitable change process occurs, which must occur accompanied by a cultural change. An enhanced collective understanding and consensus around what the new approaches will entail, needs to be at the center of this change.

Moreover, the somewhat low levels of cognitive participation suggested that efforts to identify how to match appropriate methods to the respective functions and teams within Public Health Scotland are needed. A greater effort needs to be dedicated to involving staff in the reorganization process, providing the necessary support for the employment of the new approaches.

These results need to be discussed together with several limitations. First, actors that are more open to employing complexity strategies are represented to a more significant extent, since they agreed to take part in the study. Second, our respondents are relatively advanced in their careers; hence, they might be less open to change than junior employees who will be less affected by structural change and face more manageable scale of disruption in their routine work.

Stakeholders less familiar with complexity might not be represented in the sample, but they come from the same organizational environment; hence it is reasonable to assume that if they are familiarized with the approaches, they would have opinions and reactions similar to the participants observed in our sample.

In addition, the quantitative analysis of the SWOT charts may in part reflect notes taken on the material presented in the sessions, so there may be some variations due to presenter style. All presentations followed the same format of an introductory tutorial and how the methods can be used, rather than a methodological critique. The themes emerging from the strengths and opportunities had a strong overlap with the presented material. On the other hand, the presentations did not focus on weaknesses and threats, yet the delegates did provide responses. Often, these threats and weaknesses related to structural and organizational issues, such as current ways of working and dialogue amongst evidence users.

Lastly, the choice of systems methods was limited to those where the collaborating academics had sufficient expertise to support their further use, and the session did not fully explore the range of alternative systems methods. This is a clear limitation that a larger scale project with a broader range of expertise and resources could make a useful contribution.

## Conclusion

This article introduced a framework for the assessment of the feasibility and opportunity of introducing innovation into an organizational environment. The assessment framework is applied to the appraisal of an intervention to introduce complexity approaches into the work of the network of organizations that have combined to form Public Health Scotland.

We found that there are two main obstacles to the implementation of complexity approaches: (1) a lack of a shared understanding of the key concepts in complexity and their practical implications; and (2) the fear of significant disruption to work routines and power relationships. Addressing these issues may enable complexity approaches to achieve further success, since the participants showed a considerable deal of interest.

Our assessment framework can be re-employed to assess the extent to which complexity approaches can be introduced in other health contexts (e.g., local areas, regions, countries, etc.). In general, it is particularly appropriate for the evaluation of complexity in population health, since it is built using theories widely applied in health and social care settings. However, it can also be reused to evaluate the feasibility and opportunity of introducing any other policy in a particular circumscribed situation (with appropriate adaptation to the context). For these reasons, it serves as a blueprint for the implementation of more precise assessment to be put in place before undertaking the significant work that interventions require, and it helps save time and resources that would be wasted without a careful analysis of the obstacles that may prevent success.

## Data Availability Statement

The datasets presented in this article are not readily available, and they can be partially made available because some parts are impossible to anonymize. Requests to access the datasets should be directed to mark.mccann@glasgow.ac.uk.

## Ethics Statement

The studies involving human participants were reviewed and approved by Ethics Committee College of Medical, Veterinary and Life Sciences, University of Glasgow. The patients/participants provided their written informed consent to participate in this study.

## Author Contributions

CZ and MM designed the workshop (study design) and analyzed the data. CZ wrote the first draft and MM the second. EL facilitated at the workshop (data collection), as well as data analysis and redrafting. JH facilitated the workshop (study design and data collection) as well as redrafting the article. All authors contributed to the article and approved the submitted version.

## Funding

The study was funded by the University of Glasgow EPSRC-ESRC Impact Accelerator Account. CZ, MM, and EL were supported by the United Kingdom Medical Research Council and Chief Scientist Office of the Scottish Government Health and Social Care Directorates at the MRC/CSO Social and Public Health Sciences Unit, University of Glasgow (MC_UU_12017/11, SPHSU11, MC_UU_12017/14, SPHSU14, MC_UU_00022/1, SPHSU16, MC_UU_00022/3, and SPHSU18). In addition, MM was supported by an MRC Strategic Award (MC_PC_13027); EL was supported by an MRC Skills Development Fellowship (MR/S015078/1); CZ finished the article while working at Tilburg University, Jheronimus Academy of Data Science, (NL).

## Conflict of Interest

The authors declare that the research was conducted in the absence of any commercial or financial relationships that could be construed as a potential conflict of interest.

## Publisher's Note

All claims expressed in this article are solely those of the authors and do not necessarily represent those of their affiliated organizations, or those of the publisher, the editors and the reviewers. Any product that may be evaluated in this article, or claim that may be made by its manufacturer, is not guaranteed or endorsed by the publisher.

## Footnotes

^1^Ethics approval available upon request.
