# Supplementary material for: Appraising the Implementation of Complexity Approaches Within the Public Health Sector in Scotland. An Assessment Framework for Pre-Implementation Policy Evaluation
Source: Front Public Health. 2021 Sep 22;9:653588. doi: 10.3389/fpubh.2021.653588 (PMC8492954; doi:10.3389/fpubh.2021.653588)
Supplement: Supplementary file 1 [file Data_Sheet_1.pdf]

Supplementary Material

Assessment framework - Phase One

Open Questions

Can you please introduce yourself and your role in your organisation?

Can you tell us a bit about your background and expertise in relation to Health Scotland?

What does your current job role entail? How many staff are you responsible for?

What do you understand by the following terms:

Complexity?

Systems science?

Complex intervention?

Are you familiar with these concepts in relation to public health?

If answer yes: When / where have you heard about them? What do you think about them?

e-Hit statements to rate

CONTEXT 1

National policy:

Q1.

Zero: This approach is completely at odds with current or planned national policy.

Ten: This approach is entirely compatible with current and planned national policy.

Q2.

Zero: This approach will hinder the achievement of nationally directed priorities or targets.

Ten: This approach will enable the achievement of nationally directed priorities or targets.

Local policy:

Q3.

Zero: This approach is completely at odds with current or planned local policies.

Ten: This approach is entirely compatible with all current and planned local policies (at health board level) in Scotland.

CONTEXT 2

Organisational culture:

Q4.

Zero: The organisational culture is reluctant to adopt new practice and dreads change.

Ten: The organisational culture welcomes new practice on complexity and complex systems thinking in public health and embraces change.

Q5.

Zero: The multiple staff groups likely to be involved in implementing the approach have poor working relationships and poor communication with no history of problem-solving and co-operation.

Ten: The multiple staff groups likely to be involved in implementing the new approach have good working relationships and good communication with a history of problem-solving and co-operation.

Local culture:

Q6.

Zero: There is no local sponsor, or the local sponsor is not well respected, or is likely to devote insufficient time and energy to promoting the approach.

Ten: The approach is strongly supported by a well-respected local sponsor who will provide the time and energy needed to promote the implementation.

Q7.

Zero: There are particular opinion leaders within the organisation who are likely to oppose implementation of this new practice.

Ten: There are particular opinion leaders within the organisation who are likely to support the implementation of this new practice.

Resources and risk:

Q8.

Zero: The organisation is under-resourced, and cannot fully meet the costs and additional workload resulting from the implementation of the new approach, including e.g., the training, on-going support, contingencies, publicity.

Ten: The organisation is well resourced, and can fully meet the costs and additional workload resulting from the implementation of this new approach in public health, including e.g., training, on-going support, contingencies, publicity.

Q9.

Zero: The new approach is likely to disrupt the existing allocation of resources or the formal or informal norms by which they are allocated.

Ten: The new approach will make no change to existing allocation of resources or the formal or informal norms governing allocation of resources.

Risk:

Q10.

Zero: The proposed approach is completely at odds with the organisation's existing risk management policies. Ten: The proposed approach is entirely compatible with the organisation's existing risk management policies.

Impact on public health practice:

Q11.

Zero: This new approach will disrupt public health tasks e.g. it will make them slower, introduce new tasks that are inappropriate.

Ten: This new approach will facilitate routine organisational tasks e.g. it will make them faster, or improve the effectiveness of meeting objectivess.

Q12.

Zero: There are serious concerns about the reliability, security and confidentiality of the new approach in public health.

Ten: The new approach is entirely credible in terms of security, confidentiality and reliability.

Ease of use / understanding of how this will improve public health:

Q13.

Zero The new approach is difficult to understand/ does not do what it is supposed to do.

Ten: The new approach is easy to understand and fit for purpose.

Effectiveness and cost –effectiveness:

Q14.

Zero: This new approach has not been evaluated nor demonstrated to improve health outcomes / achieve research outcomes in a cost –effective manner.

Ten: This new form of practice has been well evaluated and has been demonstrated to improve health outcomes / achieve research outcomes in a cost –effective manner.

Impact on work and workflow:

Q15.

Zero: There is likely to be a period of considerable increased workload during the implementation.

Ten: There is unlikely to be any period of increased workload during the implementation.

Q16.

Zero: This new approach will disrupt existing work patterns and make them less efficient.

Ten: This new approach will increase the efficiency of current work patterns.

Q17.

Zero: This new approach will substantially disrupt current divisions of labour between staff groups.

Ten: This intervention is completely compatible with current divisions of labour.

Education and training:

Q18.

Zero: All staff who engage in complex interventions in Public Health Scotland will need very substantial training prior to use.

Ten: No training will be needed by any staff group prior to implementation of the new approach.

Relationships between different professional groups:

Q19.

Zero: The proposed new approach will substantially disrupt existing power relationships between different staff groups.

Ten: The proposed new approach is entirely compatible with existing power relationships between different staff groups.

Q20.

Zero: This new approach will undermine confidence about each other’s expertise and performance between different professional groups in Public Health.

Ten: This new approach will increase confidence about each other’s expertise and performance between different professional groups in Public Health.

Q21.

Zero: Responsibility and accountability are not aligned, i.e., different staff groups have responsibility for how the approach is used, and accountability for the outcome of the intervention.

Ten: Responsibility and Accountability are fully aligned, i.e., one staff group is both responsible for how the approach is used and

Second table configuration tasks analysis:

Workshop tasks	Task structure	Table SNA	Table ABM	Table System Thinking and Mapping	Table Health Scotland
**Six Cohering Questions**	What are we trying to achieve?	Project level - specific	Strategic level -- specific	Strategic level -- broad	No output
Answer categorized as Project VS Strategic	What do we think it needs to get done to achieve it?	Project level - specific	Strategic level -- specific	Strategic level -- specific	No output
Broad VS Specific	Who is going to get those things done?	Project level - specific	Project level - specific	No output	No output
	Who benefits or is impacted by trying to achieve it?	Project level - specific	No output	Strategic level -- broad	No output
	Who is in charge?	Project level - specific	No output	Project level - specific	No output
	What constraints apply?	Strategic level -- specific	No output	Strategic level -- specific	No output
**Laddering Thematic analysis**	Focal question – each group choses one	In what was might Health Scotland use complexity methods to reduce inequalities?	In what ways might local authorities develop an open space strategy?	In what ways might system thinking deal with obesity in Glasgow?	In what ways might a research development group improve system practice in Public Health Scotland?
	Why would we do this?	Theoretical, methodological and moral reasons	Legal and moral reasons	Methodological reasons	Methodological and moral reasons
	How would we do this?	Collaborative approaches	Collaborative approaches Integration of old and new methods	Legislation, laddered down into “how to inform legislation?”	Improving reflective practice
**SWOT charts**	Ratio positive/negative comments in each table	1.36	0.74	0.69	0.83
	Count by topic Total Count Strength 36 Weakness 39 Opportunities 23 Threats 32	Strength 14 Weakness 10 Opportunities 5 Threats 4	Strength 12 Weakness 7 Opportunities 8 Threats 11	Strength 9 Weakness 11 Opportunities 9 Threats 15	

Thematic Analysis Pigs in the Middle Diagrams

Theme	SNA	ABM	System Thinking and Mapping	System Practice within Health Scotland
**Private Actors**	• Marketing Companies<br>• Private Sectors	• Data producers<br>• IT developers	• Pharmaceuticals companies<br>• Private Sector<br>• Consultancy	• Support for marketing<br>• Support for business excellence<br>• Support for IT
**Government and Policy Makers**	• Government<br>• Policy makers (local vs nationals)	• National Government<br>• Local authorities	• Scottish Government<br>• UK Government<br>• Local Government<br>• Community planning partnership	• Scottish Government<br>• Welfare reform<br>• Local authorities
**Other agencies/networks**	• Professional Networks<br>• Other agencies/sectors<br>• Law/Police	• Delivery parties<br>• Other application areas (engineering, defence)	• Consultancy<br>• Third Sector<br>• Police<br>• Fire Brigade<br>• Volunteer sector	• Third Sector<br>• Workforce learning and empowerment
**Public Health actors**	• Public Health Agencies	• Public Health Scotland	• Public Health Scotland<br>• Health Care Providers	• Senior executive leadership<br>• Public Health Intelligence
**Population**	• Research Participants	• Public	• Patients<br>• Community safety partnership<br>• Public	• Neighbourhoods
**Expert domain**	• Academic Users	• People who know what it is and value it<br>• ABM geeks	• Academics	• Public Health Science<br>• Medical Research Council
**Implementation**				• Complex Intervention<br>• Strategic Planning<br>• Programme teams

Pig in the middle with perspectives

Theme	SNA	Perspectives on SNA	ABM	Perspectives on ABM	System Thinking and Mapping	Perspectives on ABM	System Practice within Health Scotland	Perspectives on Systems Practice
**Private Actors**	•Marketing Companies<br>•Private Sector	Money<br>Improve Practice/optimise output/ Facebook, Twitter	•Data producers<br>•IT developers	work to find new applications for their data<br>income	•Pharmaceuticals companies<br>•Private Sector<br>•Consultancy	change in demand<br>money		
**Government and Policy Makers**	•Government<br>•Policy makers (local vs nationals)	Data Protection/ Election<br>understanding/informing policy	•National Government<br>•Local authorities	Buzz Word<br>Don't know it exists	•Scottish Government<br>•UK Government<br>•Local Government<br>•Community planning partnership	jargon, resource allocation, operational optimization<br>Strategic Vision<br>No response<br>No response	•Scottish Government<br>•Welfare reform<br>•Local authorities	
**Other agencies/networks**	•Professional Networks<br>•Other agencies/sectors<br>•Law/Police	consultancy/citation network/public health nutrition network<br>"me too please"/"don't forget our sector" understanding criminal bx	•Delivery parties<br>•Other application areas (engineering, defence)	No response<br>great	•Consultancy<br>•Third Sector<br>•Police<br>•Fire Brigade<br>•Volunteer sector	Money<br>No response<br>No response<br>No response<br>No response	•Third Sector<br>•Workforce learning and empowerment	How to build capacity<br>without confusing people?
**Public Health actors**	•Public Health Agencies		•Public Health Scotland		•Public Health Scotland	visualising complex problems	•Senior executive leadership	

		awareness/applied use		don't know what it is, emperors new clothes, more evidence needed (case studies)	•Health Care Providers	solution for planning action going forward	•Public Health Intelligence	
							•Support function in PHS: marketing<br>•Support: business excellence<br>Support<br>•Support: IT	Linear, project focus<br>Want to predict outputs What accountability incentives are there for non linear approaches?
**Population**	•Research Participants	Better health, ethics, consumers	•Public	What to it?	•Patients<br>•Community safety partnership<br>•Public		•Neighbourhoods	
**Expert domain**	•Academic Users	career progress/theory testing/collabs	•People who know what it is and value it<br>•ABM geeks	improvement on current methods<br>a tool to help decision making, publications, income, [NA]	•Academics		•Public Health Science<br>•Medical Research Council	Which models are most useful?<br>Methods research
**Implementation**							•Complex Intervention<br>•Strategic Planning<br>•Programme teams	

Thematic Analysis Context Diagram by category

Circle Number (from inner to outer)	Circle Name	SNA	ABM	System Thinking and Mapping	System Practice within HS
0	**Topic**	NHS Health Scotland	One person in the Group<br>Manager of *** team within ISD?/Organisation?	Population Health with System Thinking	Health equity directorate
1	**Factors under the stakeholders' control**	domain specific knowledge,<br><br>relationship training,<br><br>budget priorities,<br><br>staff	Public Health Scotland<br><br>SPHSU	People, academics, Scottish government, policy makers locally and nationally	learning culture<br><br>training promotion<br><br>management and accountability<br><br>recruitment<br><br>Leadership
2	**Factors stakeholders can influence but aren't directly in control**	method development<br><br>data collection<br><br>political priorities<br><br>information<br><br>government	holders of the data<br><br>existing tools and policies<br>local authority planners<br><br>domain experts<br><br>local experts<br>National Partners<br><br>time	publications<br><br>UK government	system supportive interactions<br><br>PHS functions public services<br><br>working relationships public health teams
3	**Immediate environment that constraints stakeholders' behaviour and choices**	alternative approaches<br><br>evidence<br><br>skills acceptability	availability of data evidence<br><br>Skills<br><br>ABM implementers availability		Public health reform "whole system approach"

			elected members<br>local policies<br><br>time<br><br>money and opportunity costs	 <br><br><br><br><br><br><br><br><br><br><br>industry	local authority funding<br>public health priorities<br><br><br><br><br><br><br><br><br><br><br>3rd sector<br><br>CPP<br><br>P.S.
4	**Wider context that determines what does and does not occur**				Uncertainty around system method<br><br>time<br><br>resources<br><br>socialization.

Ladders

Group	Chosen Question	Why?	How?
SNA	In what ways might Health Scotland use complexity methods to reduce health inequalities?	• limits of current methods used<br>• To reduce health inequalities<br>• understanding mechanisms	• Stakeholders' engagement
ABM	In what ways might Local Authorities develop an open space strategy?	• To meet Legal requirement<br>• To combat Climate change<br>• So Children and families can have fun<br>• To improve health<br>• To reduce health inequalities<br>• To improve community cohesion<br>• To improve attainment	• Place standard tool<br>• Community councils<br>• Community Planning partnership<br>a. Regular meeting<br>b. Understanding Findings and learning from previous experience<br>• Geographic information system and Agent Based Models<br>• Other local strategies/policies
System Thinking and Mapping	In what ways might System thinking deal with obesity in Glasgow?	• To understand context environment<br>• To provide more information about the problem<br>• To involve multiple perspectives<br>a. How is it currently done? Via CPP, local team?	• Legislate? Map out licensing decisions to be made<br>a. In what ways might Glasgow licence food outlets differently?<br>• Address the food system and understand dynamics<br>a. In what ways might system thinking in Glasgow deal with food system dynamics?<br>• Produce new advanced summary outputs
System Practice within Health Scotland	In what ways might a Research Development Group improve system practice in Public Health Scotland?	• To improve relevance and resilience of public health practice<br>• To improve quality of system science approaches<br>• To improve robustness of decision making	• Improving reflective practice

SWOT by topic

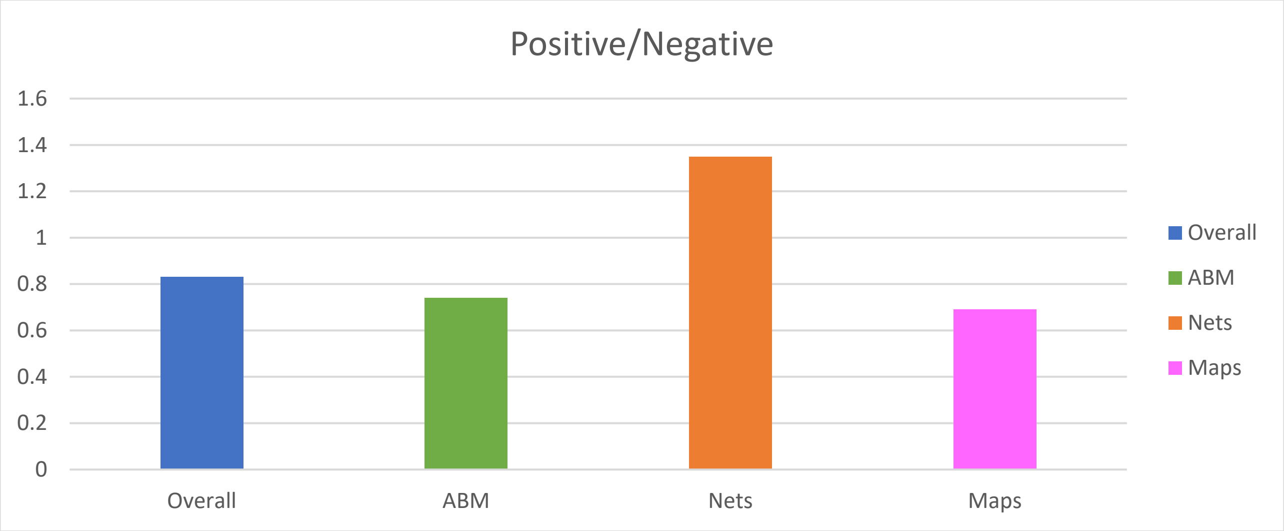

SWOT by table

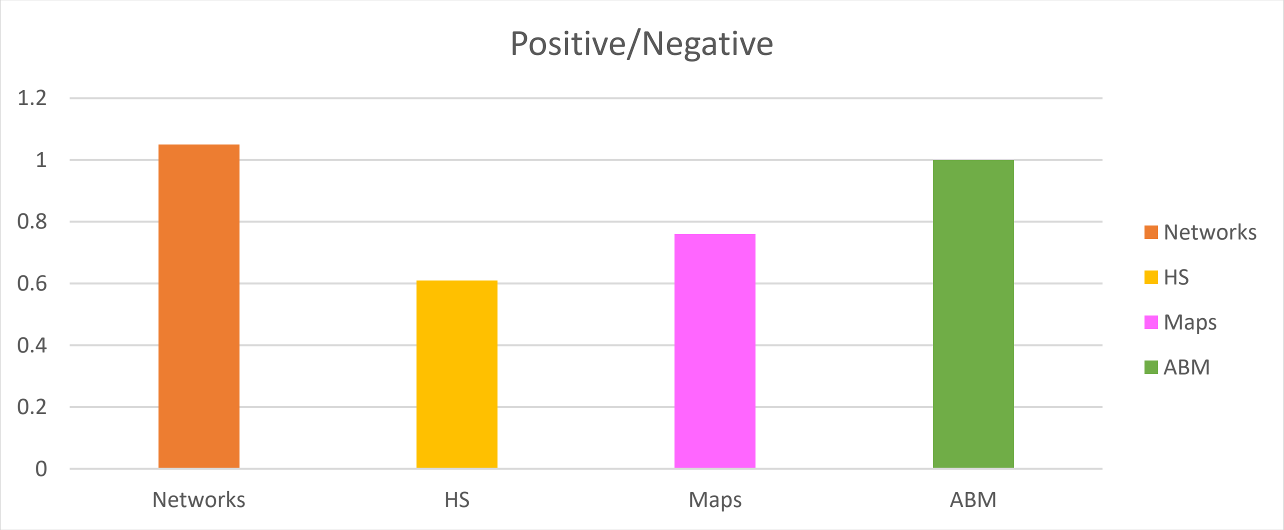

SWOT thematic analysis

SWOTs	SNA	ABM	System Thinking and Mapping
**Strengths**	• SNA has a larger explanatory power compared to other methods (visualization, intuitive)<br>• It can be applied to achieve a certain goal specifically (influence, increase understanding, model structural power)<br>• It enhances the statistician toolkit (relational data, combination with other approaches).	• Looking at causal mechanisms<br>• Ability to simulate interventions<br>• Identify gaps<br>• Problem framing and theory building	• Collaboration (foster collaboration between co-workers and communities to map out the problems/scenarios)<br>• Explanatory power (provides means to picture complexity/ single out variables).
**Weaknesses**	• Is the method useful for population health?<br>• When is it good to use SNA (is world too complex to reduce it to a network?)<br>• Difficult to use/misuse (possibility of biases)	• Risks around assumptions<br>• Fit with current ways of working<br>• Expertise, resources and siloed knowledge<br>• Understandability of the approach	• Difficulties in usage (can be unclear, affected by the designers and variable selection, hard to read, hard to sell)<br>• Lack of goals (a map is not addressing a goal, how to use it to prioritise/change?)<br>• Individual vs systems (a system visualisation might not clarify how to help one individual)
**Opportunities**	• Try unexplored research in Public Health (ego nets, network interventions)<br>• Advantages (observe response to interventions over time, show other existing networks)	• Exploring assumptions & mechanisms<br>• Ability to simulate interventions<br>• Change in policy and planning focus<br>• Link to other methods	• Collaboration (make people work together)<br>• Method innovation (hard and soft, disentangle problems, discovers new variables, evaluates, disseminates)<br>• Good to try in the new body in Scotland
**Threats**	• Misuse (bias in connecting actors/ untraced overlaps between different networks)<br>• Research Design (individual vs network observation, its ethics/ effects of interventions on different overlapping networks).	• Risks around assumptions<br>• Representing uncertainty<br>• Dialogue & disbelief among evidence users	• Is the method up to the task? (Different views, communicating complexity, misinterpretation, structural uncertainty, difficult to read, how to check for assumptions and uncertainty, not parsimonious)<br>• Usability (difficulty in drawing, time consuming, boundaries of the system, competence of researchers)<br>• For what do we need maps? (What can be achieved? which questions can we answer? Useful for interventions?)

Six Questions

6 Questions	SNA	ABM	System Thinking and Mapping	System Practice within Health Scotland
**What are we trying to achieve?**	better understanding of weather network created for a certain project is achieving its goals	• Support the use of system practice and system methodologies to improve population health, as appropriate<br>• To enable the use of system thinking in PHS<br>• Clarify existing state and practices and agreeing terminologies	The normalization (application) of system thinking in the new public health system	NA
**What do we think needs to get done to achieve it?**	1. Collect relational data (with attributes) on the topic<br>2. Apply SNA to the sample<br>3. See whether results make sense	• Build toolkits/frameworks for using system thinking<br>• Need to implement this in a "system" way to support adoption<br>• Develop a way to reflect and evaluate how system thinking and methodologies are being used (culture change in a state of flux, community of practice).	• A clear articulation of what it is<br>• Demonstration of value, promising practice in early adopters<br>• Show how it can build (add value) to current thinking/practice<br>• Acceptance of new knowledge<br>• Understand its strength/limitation/ when to use it<br>• Leadership<br>• Training and development<br>• Learning culture: it's ok to make mistakes, improvements<br>• Understanding our partners/collaboration.	NA
**Who is going to get those things done?**	• IT departments<br>• Analysts (academics/…)<br>• Stakeholders	• Academics<br>• Evangelists within the organization (with commitment and endorsement to support them (they already have jobs)	NA	NA
**Who benefits or is impacted by trying to achieve it?**	• level1: people in the network<br>• level2: population	NA	People of Scotland	NA

**Who is in charge?**	• The project team and the health equity directorate.	NA	CEO or dedicated officer of PHS	NA
**What Constraints apply?**	• Money<br>• Capacity<br>• Time<br>• Knowledge/Skills<br>• Responsiveness of Network Members<br>• Ethics (data,…)	NA	• Competing priorities<br>• Skill shortage<br>• Time<br>• Money<br>• Cynicism	NA
